# CACNA1B (Ca_v_2.2) Overexpression and Its Association with Clinicopathologic Characteristics and Unfavorable Prognosis in Non-Small Cell Lung Cancer

**DOI:** 10.1155/2017/6136401

**Published:** 2017-01-03

**Authors:** Xiaoyu Zhou, Wei Wang, Shu Zhang, Xudong Wang, Zhiyuan Tang, Jun Gu, Jun Li, Jianan Huang

**Affiliations:** ^1^Department of Respiratory Medicine, The First Affiliated Hospital of Soochow University, Suzhou, China; ^2^Department of Respiratory Medicine, Affiliated Hospital of Nantong University, Nantong, Jiangsu 226001, China; ^3^Department of Pathology, Affiliated Hospital of Nantong University, Nantong, Jiangsu 226001, China; ^4^Department of Laboratory Medicine, Affiliated Hospital of Nantong University, Nantong, Jiangsu 226001, China

## Abstract

CACNA1B (Ca_v_2.2) encodes an N-type voltage-gated calcium channel (VGCC) ubiquitously expressed in brain and peripheral nervous system that is important for regulating neuropathic pain. Because intracellular calcium concentration is a key player in cell proliferation and apoptosis, VGCCs are implicated in tumorigenesis. Recent studies have identified CACNA1B (Ca_v_2.2) being overexpressed in prostate and breast cancer tissues when compared to adjacent normal tissues; however, its role in non-small cell lung cancer (NSCLC) has not been investigated. In this study, we determined the mRNA and protein expression of CACNA1B (Ca_v_2.2) in NSCLC tumorous and adjacent nontumorous tissues by quantitative reverse transcription PCR (qRT-PCR) and tissue microarray immunohistochemistry analysis (TMA-IHC), respectively. CACNA1B (Ca_v_2.2) protein expressions in tumorous tissues were correlated with NSCLC patients' clinical characteristics and overall survival. CACNA1B (Ca_v_2.2) mRNA and protein expression levels were higher in NSCLC tumorous tissues than in nontumorous tissues. High CACNA1B (Ca_v_2.2) protein expression was associated with higher TNM stages, and CACNA1B (Ca_v_2.2) protein expression is an independent prognostic marker in NSCLC. Based on our results, we conclude that CACNA1B (Ca_v_2.2) plays a role in NSCLC development and progression. Elucidating the underlying mechanism may help design novel treatment by specifically targeting the calcium regulation pathway for NSCLC, a devastating disease with increasing incidence and mortality in China.

## 1. Introduction

Primary lung cancer remains the leading cause of cancer death worldwide and in China [1–3]. It is estimated that 605,900 patients were diagnosed and 486,600 patients died of lung cancer in 2010 in China [4, 5]. Lung cancer incidence and mortality are higher in men and urban areas than those in women and rural areas, and it is estimated that air pollution will replace smoking as the primary cause of lung cancer in China by 2020 [4]. Non-small cell lung cancer (NSCLC) accounts for over 80% of these lung cancer cases and includes the following histologic types: adenocarcinoma, squamous cell carcinoma, large cell carcinoma, and mixed histologies [6, 7]. About a quarter to a third of NSCLC patients are diagnosed with stage I or II disease, which allows surgical resection with curative intent [8]. However, despite a complete and presumably curative resection, approximately 40–50% of patients with resected NSCLC die of recurrent disease [9]. Molecular prognostic markers are needed to identify subset of patients that would benefit from aggressive treatment after surgical resection [10].

Calcium (Ca^2+^) is a key mediator of signaling transduction pathways regulating cell cycle, cell proliferation, and cell death [11–13]. Ca^2+^ can regulate the activities of many intracellular enzymes including kinases and phosphatases, and slight variations in Ca^2+^ level and distribution could activate or inhibit specific cell functions, thus intracellular Ca^2+^ alteration is associated with several pathological conditions, including cancer [14]. A number of mechanisms are used to precisely regulate the intracellular Ca^2+^ concentration, including active transporting Ca^2+^ out of the cell, storing Ca^2+^ in the endoplasmic reticulum (ER).

The voltage-gated calcium channels (VGCCs) are main regulators of intracellular Ca^2+^ level. Overexpression of various VGCC members has been detected in various cancer types, including leukemia [15], breast [16], prostate [17], ovarian [18], and lung cancer [15]. CACNA1B (Ca_v_2.2) is the only member in N-type VGCC family, and CACNA1B (Ca_v_2.2) overexpression has been detected in breast and prostate cancer [15], and it represents a novel therapeutic target for the treatment of breast and prostate cancer. However, its role in NSCLC is unknown.

In the current study, we determined both mRNA and protein expression of CACNA1B (Ca_v_2.2) in NSCLC tissue samples by quantitative reverse transcription PCR (qRT-PCR) and tissue microarray immunohistochemistry analysis (TMA-IHC), respectively, and correlated to patients' clinical characteristics and overall survival.

## 2. Material and Methods

### 2.1. Human Tissue Specimens and Patient Clinical Information

A total of 164 NSCLC patients were included in the study. Twenty-four NSCLC patients consented and were enrolled before surgery, and 24 pairs matched tumorous and nontumorous fresh tissue samples were collected and frozen at the time of surgery. All samples in this study were from clinical biobank in Affiliated Hospital of Nantong University, Jiangsu Province, China. In addition, 140 NSCLC patients provided 140 pairs matched tumorous and nontumorous formalin-fixed paraffin-embedded (FFPE) tissue blocks. Clinical characteristics were obtained from patients' medical records. In the current study, the normal control samples are defined as adjacent nontumorous tissue samples. The study protocol was approved by the Human Research Ethics Committee of the Affiliated Hospital of Nantong University, Jiangsu, China.

### 2.2. CACNA1B (Ca_v_2.2) mRNA and Protein Expression and Statistical Analysis

CACNA1B (Ca_v_2.2) mRNA level was determined by quantitative reverse transcription PCR (qRT-PCR) using relative quantification method by normalizing to the housekeeping gene GAPDH [19]. The primers used are as follows: CACNA1B (Ca_v_2.2) forward primer (5′-AAC ATT CTG GAC TTC ATT-3′) and CACNA1B (Ca_v_2.2) reverse primer (5′-AGA GAC TTG ATG GTA TTG-3′) and GAPDH forward primer (5′-TGC ACC ACC AAC TGC TTA GC-3′) and GAPDH reverse primer (5′-GGC ATG GAC TGT GGT CAT GAG-3′). CACNA1B (Ca_v_2.2) protein expression in tissue blocks was determined using tissue microarray immunohistochemistry (TMA-IHC) [20]. Rabbit polyclonal anti-human CACNA1B (Ca_v_2.2) antibody was used (dilution 1 : 500, ab121193, Abcam, USA). The CACNA1B (Ca_v_2.2) IHC data were scored using the semiquantitative H-score method taking into account both the staining intensity and the percentage of cells at that intensity [21], ranging from 0 to 300. Subsequently, the continuous CACNA1B (Ca_v_2.2) protein expression data were converted into dichotic data (low versus high) using specific cutoff values, which were selected to be significant in terms of overall survival (OS) using the X-tile software program (The Rimm Lab at Yale University; http://medicine.yale.edu/lab/rimm/research/software.aspx) [22, 23]. In the current study, the cutoff was 90: score 0–90 was considered low expression while 100–300 was considered high expression.

Statistical analysis was performed as described before [24]. Student's *t*-test was used to compare CACNA1B (Ca_v_2.2) mRNA and protein expression between tumorous and nontumorous tissue samples. Pearson *χ*^2^ tests were performed to determine the correlation between CACNA1B (Ca_v_2.2) expression and clinicopathologic parameters. Univariate and multivariate Cox regression models were used to identify prognostic factors. Kaplan–Meier method was used to calculate survival curves. For all analyses, a *P* value < 0.05 was regarded as statistically significant. Data were analyzed using SPSS 20 statistics software (SPSS Inc., Chicago, IL, USA) and STATA 12.0 (StataCorp, College Station, TX, USA).

## 3. Results

### 3.1. CACNA1B (Ca_v_2.2) mRNA Level Was Significantly Higher in NSCLC Tumorous Tissues than in Adjacent Nontumorous Tissues

**Figure 1:**
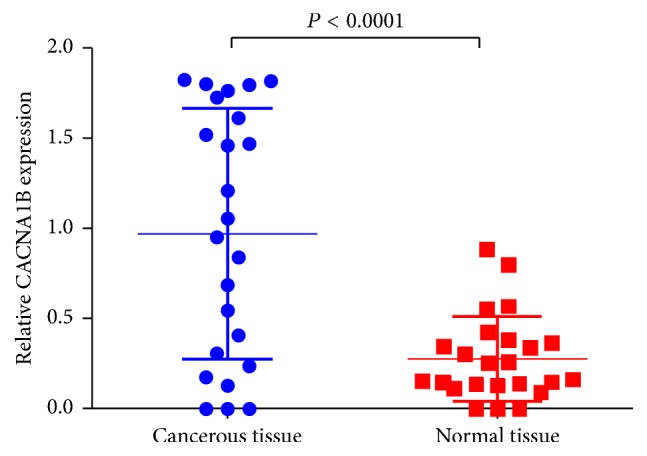
CACNA1B (Ca_v_2.2) mRNA level was significantly higher in NSCLC tumorous tissues than in adjacent nontumorous tissues. CACNA1B (Ca_v_2.2) mRNA was determined by qRT-PCR and relative quantification analysis by normalizing to GAPDH mRNA.

We determined CACNA1B (Ca_v_2.2) mRNA level in 24 pairs of fresh frozen NSCLC tumorous and adjacent nontumorous tissues. Relative CACNA1B (Ca_v_2.2) mRNA expression level was normalized to the expression of housekeeping gene GAPDH. CACNA1B (Ca_v_2.2) mRNA expression level was significantly higher in NSCLC tumorous tissues (0.97 ± 0.14) when compared to adjacent nontumorous tissues (0.28 ± 0.05) (*P* < 0.001) (Figure 1).

### 3.2. CACNA1B (Ca_v_2.2) Protein Level Was Significantly Higher in NSCLC Tumorous Tissues than in Adjacent Nontumorous Tissues

**Figure 2:**
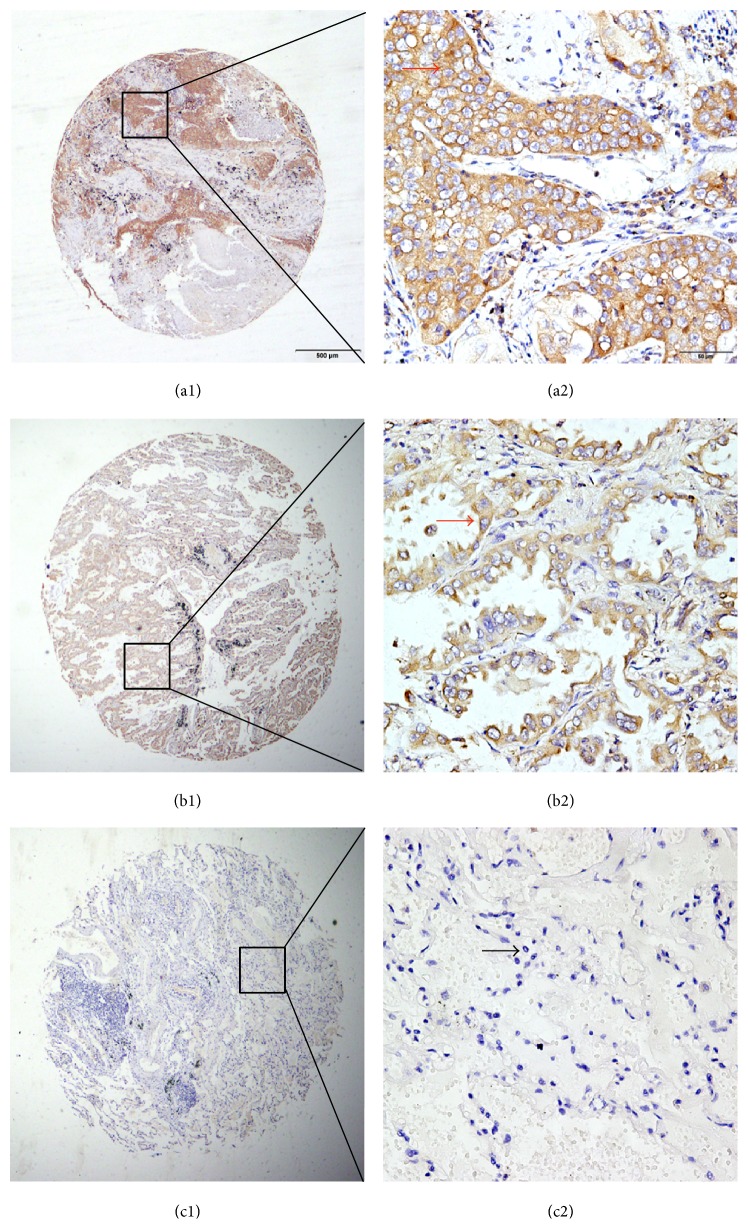
Representative immunohistochemistry (IHC) images showing expression of CACNA1B (Ca_v_2.22) in tissue microarray sections of NSCLC. (a1) and (a2) show strong positive staining in the cytoplasm of squamous cell carcinoma tissues. (b1) and (b2) show moderate positive staining in the cytoplasm of adenocarcinoma tissues. (c1) and (c2) show a negative IHC reaction in matched adjacent normal tissues. Original magnification was ×40 for (a1) and (b1) and ×400 for (a2) and (b2).

We determined CACNA1B (Ca_v_2.2) protein expression in 140 pairs matched tumorous and adjacent nontumorous archived NSCLC tissue blocks. High CACNA1B (Ca_v_2.2) expression was detected in 58.57% of tumorous tissues, with significantly higher than 41.43% detected in matched adjacent nontumorous tissues (Table 1, Pearson *χ*^2^ = 29.594, *P* < 0.001). Typical IHC staining patterns for CACNA1B (Ca_v_2.2) in NSCLC are shown in Figure 2.

### 3.3. Association of CACNA1B (Ca_v_2.2) Expression with NSCLC Clinical Characteristics

Next, we correlated CACNA1B (Ca_v_2.2) protein expression with NSCLC patients' clinical characteristics, including gender, age at diagnosis, tumor size, histopathology grading, lymph node metastasis, smoking history, and TNM stage. High CACNA1B (Ca_v_2.2) protein expression was significantly associated with gender (Pearson *χ*^2^ = 5.108, *P* = 0.018), tumor size (Pearson *χ*^2^ = 9.435, *P* = 0.002), histopathology grading (Pearson *χ*^2^ = 9.222, *P* = 0.010), lymph node metastasis (Pearson *χ*^2^ = 10.541, *P* = 0.005), and smoking (Pearson *χ*^2^ = 4.415, *P* = 0.029) (Table 1).

### 3.4. High CACNA1B (Ca_v_2.2) Expression Predicts Poor Overall Survival in NSCLC Patients

**Figure 3:**
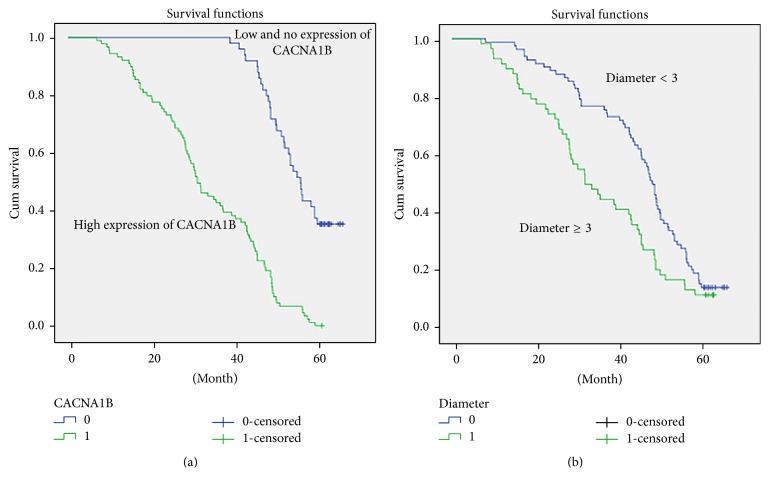
Survival curves of NSCLC patients by the Kaplan–Meier method and the log-rank test. (a) NSCLC patients with high CACNA1B (Ca_v_2.2) expression (green line, 1) had significantly worse overall survival than NSCLC patients with low or no CACNA1B (Ca_v_2.2) expression (blue line, 0); (b) NSCLC patients with larger tumor (3 cm) (green line, 1) had significantly worse overall survival than NSCLC patients with smaller tumor (<3 cm) (blue line, 0).

Finally, we analyzed prognostic factors in NSCLC patients using both univariate and multivariate analyses. In univariate analysis, high CACNA1B (Ca_v_2.2) expression (HR, 2.701, 95% CI: 1.797–4.061; *P* < 0.001), male (HR, 1.530, 95% CI: 1.070–2.188; *P* = 0.020), large tumor size (3 cm) (HR, 2.064, 95% CI: 1.431–2.976; *P* < 0.001), high histopathology grading (HR, 0.419, 95% CI: 0.306–0.574; *P* < 0.001), lymph node metastasis (HR, 1.482, 95% CI: 1.005–2.185; *P* = 0.047), smoking (HR, 2.237, 95% CI: 1.365–3.666; *P* = 0.001), and advanced TNM stage (HR, 1.425, 95% CI: 1.108–1.833; *P* = 0.006) were significantly associated with overall survival. These significant factors were then included in the multivariate analysis. In multivariate analysis, high CACNA1B (Ca_v_2.2) expression (HR, 2.639, 95% CI: 1.699–4.099; *P* < 0.001), high histopathology grading (HR, 0.572, 95% CI: 0.392–0.837; *P* = 0.004), and smoking (HR, 2.526, 95% CI: 1.440–4.430; *P* = 0.001) remained significantly associated with poor overall survival (Table 2). Similar results were shown by the Kaplan–Meier survival curve (Figure 3).

## 4. Discussion

In the current study, we determined mRNA and protein expression levels of CACNA1B (Ca_v_2.2) in both NSCLC tumorous and adjacent nontumorous tissues. CACNA1B (Ca_v_2.2) mRNA and protein level were significantly higher in tumorous tissues than in adjacent nontumorous tissues. High CACNA1B (Ca_v_2.2) protein level was significantly associated with TNM staging. Finally, high CACNA1B (Ca_v_2.2) protein expression is an independent prognostic marker for poor overall survival in NSCLC patients.

Calcium is a key second messenger that is involved in virtually every aspect of cellular function, including cell proliferation, apoptosis, gene transcription, and angiogenesis. In normal resting cells, the cytoplasmic Ca^2+^ level is maintained at ~100 nM significantly lower than extracellular Ca^2+^ concentration and Ca^2+^ concentration in endoplasmic reticulum (ER). There is increasing evidence suggesting that an increase of intracellular Ca^2+^ concentration leads to cell growth and proliferation, while the decrease of ER Ca^2+^ concentration inhibits apoptosis [14, 25].

The precise control of intracellular free Ca^2+^ changes is essential for the proper regulation of many cellular pathways, including those important in tumorigenesis and cancer progression [14]. Ca^2+^ is a key regulator of cell cycle, thus cell proliferation [26]; excess accumulation of Ca^2+^ in mitochondria is linked to apoptosis and necrosis and reduction of Ca^2+^ in ER is associated with resistance to apoptosis [27–29]. For example, altered ER Ca^2+^ level led to cisplatin and Taxol resistance in NSCLC cell lines [30, 31], and reduced ER Ca^2+^ is a protective mechanism for prostate cancer cells escaping cell death in the absence of androgenic stimulation [32]. In addition, extracellular Ca^2+^ signaling is implicated in differentiation [33]; Ca^2+^ regulates cellular motility, thus implicated in tumor invasion and metastasis [34–36]; Ca^2+^ is a key regulator of angiogenesis signaling pathway [37]; Ca^2+^ regulates gene transcription [28] and DNA damage response pathway [38]; finally, Ca^2+^ is involved in the regulation of telomerase activity [39].

Voltage-gated calcium channels (VGCCs) are main regulators of intracellular Ca^2+^ homeostasis. There are five types of family members including L, N, T, R, and P/Q types, and their involvement in carcinogenesis has been investigated in both clinical correlational studies and in vitro functional studies. For example, clinical studies have demonstrated overexpression of CACNA1D (Ca_v_1.3) (L type), CACNA1A (Ca_v_2.1) (P/Q type), and CACNA1G (Ca_v_3.1) (T type) in lung cancer, and overexpression of CACNA1A (Ca_v_2.1) was associated with poor prognosis [15]. Mechanistically, colon cancer cells treated with calcium channel agonist induced apoptosis [40], and overexpression of CACNA2D2 in lung cancer cell lines induced apoptosis through elevating intracellular Ca^2+^ level [41, 42].

CACNA1B (Ca_v_2.2) is an N-type VGCC and expressed in the brain and the peripheral nervous system. Previous study has linked CACNA1B (Ca_v_2.2) to neuropathic pain [43, 44] and CACNA1B (Ca_v_2.2) mutation (R1389H) has been linked to myoclonus-dystonia syndrome, a rare movement disorder [45]. Very little is known about its role in carcinogenesis, except overexpression of CACNA1B (Ca_v_2.2) was detected in both prostate and breast cancer [15]. Our data suggest that CACNA1B (Ca_v_2.2) is overexpressed in NSCLC tumorous tissues when compared to adjacent nontumorous tissues, and CACNA1B (Ca_v_2.2) overexpression is also an independent prognostic marker for NSCLC. Future in vitro mechanistic studies are needed to determine whether CACNA1B (Ca_v_2.2) regulates cell proliferation, apoptosis, or chemoresistance in lung cancer cells and whether CACNA1B (Ca_v_2.2) influences the intracellular or ER Ca^2+^ levels.

Our study has several limitations. First, our study is retrospective and subject to sample selection bias, so our conclusions could not be directly extended to other populations without further validation. Second, our sample size is small so we were unable to perform the analysis by cancer histological types. Third, we did not provide a mechanism for the role of CACNA1B (Ca_v_2.2) in tumor development. It is unknown whether CACNA1B (Ca_v_2.2) expression is associated with alterations in intracellular Ca^2+^ concentration. Future in vitro studies are needed to elucidate the underlying molecular mechanism.

## 5. Conclusions

In conclusion, our study demonstrates that CACNA1B (Ca_v_2.2) plays a role in the development of NSCLC and CACNA1B (Ca_v_2.2) overexpression is an independent prognostic marker for NSCLC in Chinese population. The function of CACNA1B (Ca_v_2.2) is tightly linked to tumor intracellular Ca^2+^ concentration; targeting intracellular calcium level through VGCCs might represent a novel therapy for NSCLC.

## Figures and Tables

**Table 1:** Correlation of CACNA1B (Ca_v_2.2) expression in tumor tissues with clinicopathologic characteristics in non-small cell lung cancer (NSCLC) patients.

Clinicopathologic characteristics	*n*	CACNA1B (Ca_v_2.2)			
		Low or no expression	High expression	Pearson *χ*^2^	*P*
Total	140	58 (41.43)	82 (58.57)	29.594	*P* < 0.001^*∗*^
Gender					
Male	69	22 (31.88)	47 (68.12)	5.108	0.018^*∗*^
Female	71	36 (50.70)	35 (49.30)		
Age at diagnosis (years)					
≤60	66	26 (39.39)	40 (60.61)	2.063	0.103
>60	74	32 (43.24)	42 (56.76)		
Tumor size (cm)					
≤3	75	40 (53.33)	35 (46.67)	9.435	0.002^*∗*^
>3	65	18 (27.69)	47 (72.31)		
Histopathology grading					
Adenocarcinoma	92	45 (48.91)	47 (51.09)	9.222	0.010^*∗*^
Squamous cell carcinoma	30	11 (36.67)	19 (63.33)		
Others^a^	18	2 (11.11)	16 (88.89)		
Lymph node metastasis					
No regional lymph node metastasis	98	49 (50.00)	49 (50.00)		
Metastasis in ipsilateral peribronchial lymph nodes	22	6 (27.27)	16 (72.73)	10.541	0.005^*∗*^
Metastasis in mediastinal lymph nodes	20	3 (15.00)	17 (85.00)		
Smoking					
Smoking	20	4 (20.00)	16 (80.00)	4.415	0.029^*∗*^
No smoking	120	54 (45.00)	66 (55.00)		
Stage grouping with TNM					
Stage I	21	8 (38.10)	13 (61.90)	1.730	0.421
Stage II	46	16 (34.78)	30 (65.22)		
Stage III	73	34 (46.58)	39 (53.42)		

^*∗*^ *P* < 0.05; a, others, adenosquamous carcinoma.

**Table 2:** Univariate and multivariate analysis of prognostic factors in CACNA1B (Ca_v_2.2) for 5-year overall survival.

Characteristic	Univariate analysis			Multivariate analysis		
	HR	*P*	95% CI	HR	*P*	95% CI
CACNA1B (Ca_v_2.2) expression						
High versus low	2.701	<0.001	1.797 4.061	2.639	<0.001^*∗*^	1.699 4.099
Gender						
Male versus female	1.530	0.020	1.070 2.188	1.230	0.339	0.804 1.882
Age (years)						
≤60 versus >60	1.273	0.189	0.888 1.826			
Tumor size (cm)						
≤3 versus >3	2.064	<0.001	1.431 2.976	1.138	0.597	0.705 1.836
Histopathology grading						
Adenocarcinoma versus squamous cell carcinoma versus others^a^	0.419	<0.001	0.306 0.574	0.572	0.004^*∗*^	0.392 0.837
Lymph node metastasis						
No metastasis versus metastasis	1.482	0.047	1.005 2.185	0.995	0.985	0.575 1.721
Smoking						
No smoking versus smoking	2.237	0.001	1.365 3.666	2.526	0.001^*∗*^	1.440 4.430
TNM stage						
Stage I versus stage II versus stage III	1.425	0.006	1.108 1.833	1.349	0.147	0.900 2.024

^*∗*^ *P* < 0.05; ^a^others, adenosquamous carcinoma.

## Abbreviations

NSCLC: Non-small cell lung cancer
VGCC: Voltage-gated calcium channel
qRT-PCR: Quantitative reverse transcription polymerase chain reaction
TMA-IHC: Tissue microarray immunohistochemistry analysis
TNM: Tumor node metastasis
ER: Endoplasmic reticulum
FFPE: Formalin-fixed paraffin-embedded
OS: Overall survival
HR: Hazard ratio.
